# I do what I want: are our patients adhering to bracing recommendations? Early results of a prospective cohort study

**DOI:** 10.1007/s43390-026-01312-2

**Published:** 2026-02-26

**Authors:** Maia D. Regan, Ying Li, Grant D. Hogue, Megan E. Johnson, Jason B. Anari, Kevin M. Neal, Keith D. Baldwin

**Affiliations:** 1 Division of Orthopaedic Surgery, Children’s Hospital of Philadelphia, Perelman School of Medicine at the University of Pennsylvania, Philadelphia, PA USA; 2 Department of Orthopaedic Surgery, C.S. Mott Children’s Hospital, University of Michigan Health, Ann Arbor, MI USA; 3 Orthopedic Center, Boston Children’s Hospital, Boston, MA USA; 4 Texas Scottish Rite Hospital for Children, Dallas, TX USA; 5 Orthopedic Center, Nemours Children’s Health, Jacksonville, FL USA

**Keywords:** Adolescent idiopathic scoliosis, Bracing, Prospective registry, Non-operative care, Brace compliance

## Abstract

**Purpose:**

Bracing is the mainstay of treatment for growing adolescents affected by idiopathic scoliosis with curves between 25° and 45°. Previous randomized trials and prospective studies have indicated that duration of bracing is an important factor in preventing curve progression. We aimed to compare actual in brace time to prescribed time.

**Methods:**

We prospectively collected heat sensor data to assess bracing adherence. We aimed to assess brace prescription versus actual wear time and if any demographic or curve factors affected this relationship.

**Results:**

We identified 104 patients with minimum 6-month follow-up brace heat sensor data. Average brace prescription was 16.5 h/day, and wear time, as indicated by heat sensor, was 12.9 h. This constituted 3.6 less hours worn than prescribed (2.4–4.3; *p* < 0.001). Thoracic curves had decreased brace wear versus target compared to thoracolumbar/lumbar (T/L) curves. Curve magnitude was positively associated with total brace wear average, but was not associated with adherence versus target brace wear. Diminishing returns in brace wear times were noted with prescriptions over 16 h.

**Conclusion:**

Brace adherence averages 3.6 h less than prescribed or 78.2% of the original prescribed time. Patients with larger curves were prescribed longer brace wear but did not have better adherence versus target. Thoracic curves had less adherence compared to T/L curves. The only modifiable independent risk factor we were able to determine for greater brace wear was prescribed hours. This information can be used to counsel patients during bracing treatment.

## Introduction

Adolescent idiopathic scoliosis (AIS) is the most common spinal deformity in children aged 10–16 years. This condition affects between 1 and 3% of adolescents with a higher incidence in females [1–3]. AIS occurs in otherwise healthy individuals and has no known etiology [2]. In the last several decades, numerous studies have demonstrated that the natural history of AIS can be arrested, or at least slowed, by non-operative interventions, particularly bracing [4, 5]. Furthermore, studies have shown negative long-term prognosis with curve progression including pain, pulmonary compromise, and psychosocial effects [6–9].

Rapid curve progression in AIS typically occurs most classically during peak height velocity, the so called “adolescent growth spurt” [10]. In skeletally immature children, clinical practice typically indicates observation of curves with Cobb angles between 10° and 25°and bracing of curves with Cobb angles between 25 and 45° [11]. For adolescents in the bracing range, the primary objective of the brace is to arrest further progression of the curve [4, 5]. Previous studies have demonstrated curve progression proportional to curve size at the initiation of bracing along with skeletal growth remaining [3–5]. Braces are typically prescribed to be worn full time (16–20 h per day) until reaching skeletal maturity [3, 12]. The most classic brace is a thoracolumbar orthosis (TLSO)-type brace made from prefabricated polypropylene pelvic module with a soft foam polyethylene lining and is worn full time [13]. Heterogeneity in bracing success is thought to largely depend on adherence to the bracing regimen with a strong dose dependence noted between amount of time worn and success of the bracing treatment [3, 14, 15]. As such, patient adherence to brace wear is an essential component to its success. Standardized monitoring is necessary to accurately measure the relationship of brace wear and curve progression. Historically, patient-reported adherence was used to measure compliance outcomes. Yet, this method was subject to inaccuracy as adherence estimates often overestimated average wear time [16]. Temperature-based heat sensor technology is currently a popular method of adherence monitoring to objectively report how often the brace is worn.

Although the idea of brace adherence and its relationship to bracing success is not new, little is known about bracing adherence relative to prescribed hours of wear. We hypothesized that patients would have less hours of wear compared to the number of hours prescribed by their physician.

## Materials and methods

**Fig. 1:**
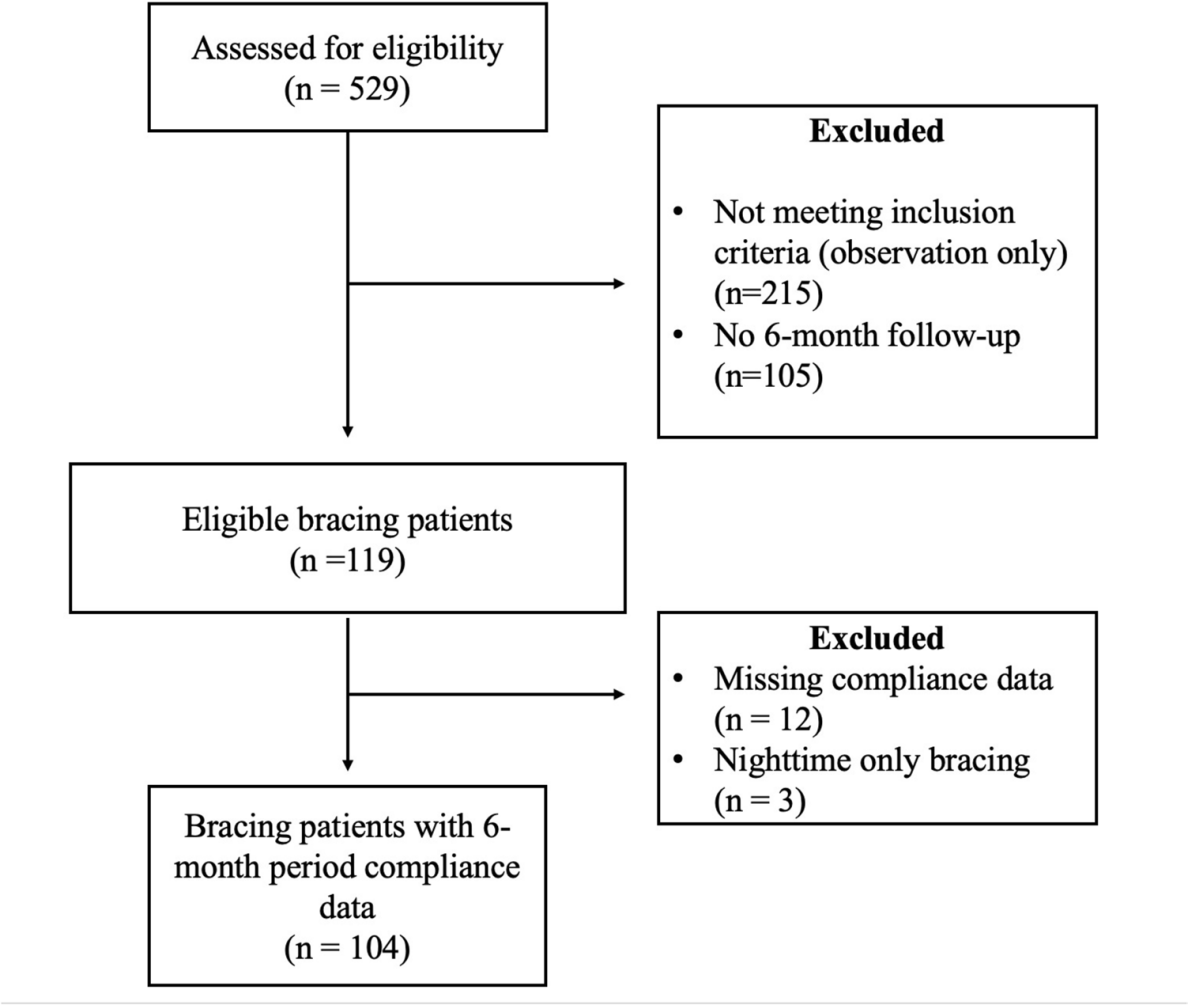
Consort diagram for inclusion in the current study

We prospectively collected patients across multiple tertiary care referral centers between 2021 and 2024 with AIS who were being non-operatively managed in braces for curves between 25° and 45° in accordance with SRS bracing protocols. Inclusion in this study consisted of having heat-sensor bracing compliance monitoring data and having a minimum of 6 months of follow-up. Each of the four institutions involved in the Scoliosis Non-Operative REgistry Study (SNORES) Group obtained approval from their respective institutional review board, and all patients/parents consented to participate. Participants were eligible for participation if they were between the ages of 10 and 16 years, had a diagnosis of adolescent idiopathic scoliosis, and obtained a hand bone age X-ray with a Sanders bone age less than 8 at initial presentation. For this study, patients who were undergoing observation or nighttime-only bracing for their AIS were excluded (Fig. 1).

Posteroanterior radiographs were evaluated at the initiation of bracing and at all subsequent 6-month follow-up visits. Radiographs were evaluated for curve location and major curve Cobb angle. Thoracic curves were established by an apex between T3 and the T11–T12 disc, while thoracolumbar/lumbar curves were classified by an apex between T12 and L4. Baseline demographics of sex, age at the index visit, race, and ethnicity were obtained. Bracing compliance data were collected including prescribed brace wear time, average brace wear per day in the last month, in the last 6 months, and since prescribed.

Adherence data were collected for each patient treated with a brace via heat sensor technology. The heat sensor gathers adherence data that is averaged over the course of treatment. The iButton (Boston Orthotics & Prosthetics, Stoughton, MA) records hours of brace wear using a heat sensor technology that transfers data via the associated Boston Sensor App or directly from the button. Data were downloaded from the iButton by clinical staff at each follow-up visit. iButton measurement extraction for scoliotic bracing is standard of care at each site in the consortium. Prescribed time for each patient was abstracted from the medical record, and total adherence was calculated by the formula: Adherence = Brace wear time–Prescribed time.

Standard descriptive summary statistics were reported, including valid percentages for categorical variables and means and standard deviations for continuous variables. Univariate analyses were used to compare between subgroups. Spearman rank correlation coefficient was used to evaluate the effect of curve magnitude on brace wear. Logistic regression was used to evaluate for predictors of brace wear. All tests were two-tailed, and significance was set at *α* = 0.05. All statistical testing was performed using SPSS Version 28 (SPSS Inc, Chicago, IL).

## Results

A total of 104 patients (23% male and 77% female) with a total of 163 individual 6-month encounters for brace compliance were identified. 95.2% of patients were prescribed a rigid TLSO (Boston) and 4.8% were a RIGO Cheneau style brace. The average brace prescription was 16.5 h (range 12–23 h). Fifty-three percent of the curves were primarily thoracolumbar/lumbar (TL/L) and 47% were primarily thoracic (T). Thoracic curves had a high TLSO-style brace, while TL/L curves had a low profile TLSO. Fifty-two percent of patients had open or closing triradiate cartilage, whereas 48% had closed triradiates. Sanders bone age breakdown was as follows: 13.4% 1 or 2, 63.8% 3 or 4, 20.1% 5 or 6, and 2.5% 7. Mean Cobb angle at the initiation of bracing was 28.9° (±7.1°). Average hours in the brace identified by the heat sensor for all patients during the entire follow-up was 12.9 h (± 4.6 h) (Table 1).

**Table 1:** Patient demographics and baseline characteristics

		*n* (%)
Sex		
	Female	80 (77)
	Male	24 (23)
Main curve location		
	Thoracic	49 (47)
	Thoracolumbar/Lumbar	55 (53)
Triradiate cartilage		
	Open/Closing	54 (52)
	Closed	50 (48)
Sanders bone age		
	1 or 2	14 (13.4)
	3 or 4	66 (63.8)
	5 or 6	21 (20.1)
	7	3 (2.5)
Initial Cobb angle	28.9 ± 7.1	
Average brace prescription	16.5 ±	
Average hours in brace (6 months)	12.9 ± 4.6	

**Fig. 2:**
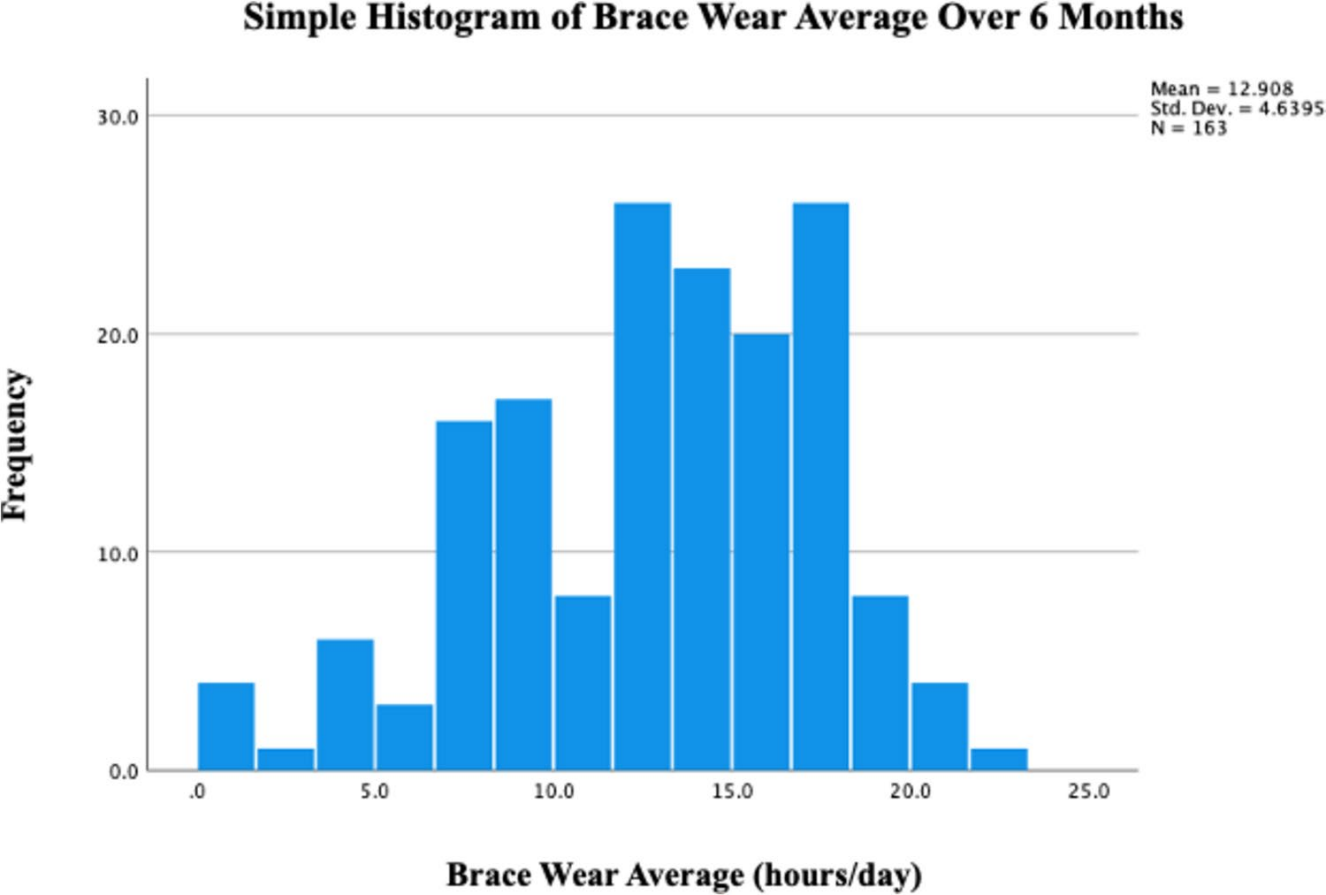
Simple histography of average brace wear over 6-month period

**Fig. 3:**
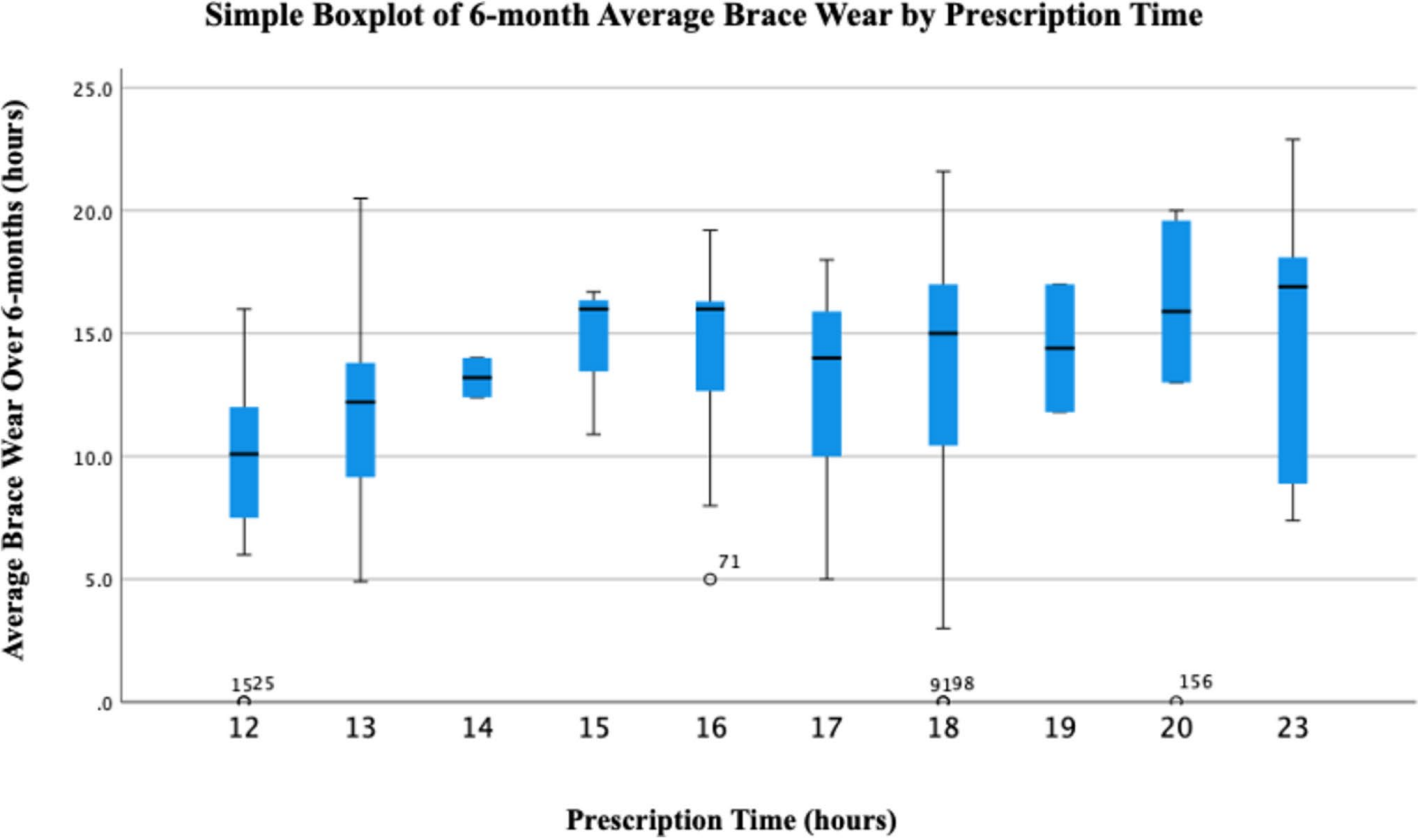
Simple boxplot of 6-month average brace wear by prescription time

**Fig. 4:**
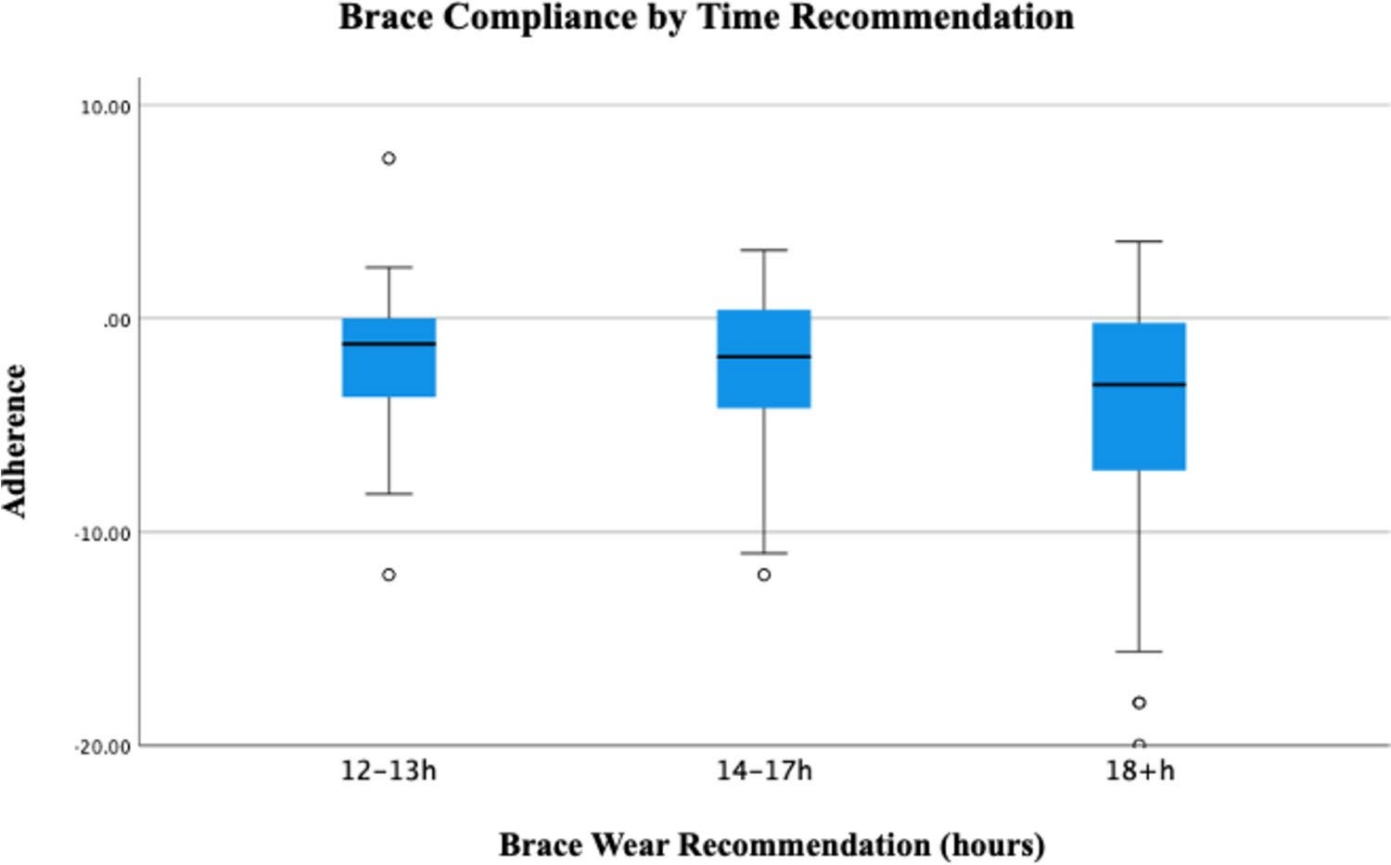
Simple boxplot of brace compliance by recommended bracing time

In the final month of a 6-month brace period, the brace was worn on average 12.7 h compared to the average 16.5 h prescribed. This constitutes 3.8 h less wear than prescribed (*p* < 0.001). When considering the entire 6-month period, the brace was worn on average 12.9 h per day compared to the 16.5 h prescribed time constituting a difference of 3.6 h less wear time than prescribed (*p* < 0.001) (Figs. 2 and 3). In addition, we identified a trend in which longer brace recommendations resulted in greater deviation from the recommended brace wear when considering specific brace wear recommendations. Patients who were recommended a brace for 12–13 h wore the brace on average 3.4 h less than recommended, whereas patients recommended 14–17 h wore the brace 4 h less than recommended. Patients prescribed for 18 or more hours wore the brace 5 h less than recommended (Fig. 4).

Furthermore, we stratified brace wear in hours per day by <12 h and ≥12 h to assess a threshold for successful treatment. We found that 86.7% of patients who wore the brace for 12 or more hours per day had a successful outcome defined by a curve progression <6° compared to 76.6% of patients who wore the brace <12 h per day (*p* = 0.116). Logistic regression analysis demonstrated that Cobb angle upon initiation of bracing, prescribed time in a brace, age, and curve location were independent predictors of brace wear greater than 12 h. Overall, 65.5% of patients wore the brace longer than 12 h. When looking at our raw data unadjusted for non-modifiable factors, prescribing the brace for 12 h resulted in a baseline 31% adherence with at least 12 h of wear. A prescription for 16 h of bracing resulted in 77.6% adherence to wearing the brace at least 12 h. Finally, a prescription of 20 h or more resulted in 72.7% adherence to 12 or more hours of brace wear.

In terms of risk factors for adherence other than hours prescribed, there was no significant difference in adherence between males and females (*p* = 0.582). Similarly, skeletal maturity was not found to influence adherence either by triradiate score (*p* = 0.071), Risser stage (*p* = 0.722), or Sanders bone age (*p* = 0.644). Chronologic age was also not found to be a risk factor for adherence (*p* = 0.151).

Patients with thoracic curves tended to struggle more with compliance, on average wearing the brace for 4.2 h less than prescribed, whereas patients with predominantly thoracolumbar/lumbar curves had less issues with an average 1.8 h less wear than prescribed (mean difference = 1.96 h; *p* = 0.007). Moreover, curve magnitude was positively associated with total brace wear average over 6 months (Rho = 0.314, *p* < 0.001) but was not associated with adherence versus target brace wear (actual brace wear versus prescribed brace wear) (Rho = 0.080, *p* = 0.334).

## Discussion

Few studies exist in the literature objectively measuring bracing adherence in patients with AIS. Historically, bracing compliance has been recorded by patient report. However, numerous studies have shown inconsistencies in reported time and actual brace wear [17–19]. Thus, the heterogeneity in available data makes it difficult to evaluate brace efficacy in AIS. To rectify this issue, several groups have developed objective adherence measures, yet few studies exist assessing this relationship with an objective measure of brace wear such as a heat sensor monitor [18, 20, 21]. Furthermore, past studies have been limited by small sample size resulting in poor generalizability of the data.

A recent study by Fregna et al. who similarly used a heat sensor iButton for systematic data collection of brace compliance demonstrated high adherence to brace wear. In contrast to our results, these investigators revealed two crucial determinants in bracing compliance, gender and both chronologic and bone age [17]. Females demonstrated significantly higher adherence to brace recommendations than males, consistent with prior smaller studies [19, 22, 23]. Furthermore, their study redemonstrated a 4% difference in compliance favoring younger patients. We did not demonstrate such a significant correlation between age and brace adherence consistent with the findings of Seleviviene et al. who examined the psychosocial determinants of brace adherence [22].

The variation in these findings highlights the need for further investigation to establish the role of age and brace compliance in AIS. Our prospective, multicenter group aims to further explore these relationships as we continue to enroll patients. Additional efforts investigating the underlying mechanisms for gender and age disparities across studies are warranted to better tailor brace recommendations for patients. Our finding that there were no differences in brace wear hours between males and females must be considered with the caveat that only 24 males compared to 80 females were included in our sample. Additional longitudinal data may provide more insight on this, as adherence may change in a more longitudinal fashion.

Several factors may affect brace wear adherence in patients with AIS. Our study demonstrated that thoracic curves wore the brace less than thoracolumbar/lumbar curves. Thus, the issue of comfort raises concern in patient adherence. Psychological factors influencing brace compliance have previously been explored and demonstrated physical comfort of the brace and perception of appearance as factors affecting brace wear [24]. Larger magnitude curves tended to wear the brace longer consistent with the association of greater prescription time for larger curves (Rho = 0.238; *p* = 0.003). However, we did not appreciate a difference in curve magnitude and adherence rate (worn-prescribed).

Tamemitsu et al. did not demonstrate any correlation between prescribed regimen and brace adherence. However, the prior study demonstrated higher adherence in younger patients [19]. Fregna’s study uncovered a trend towards significance in the relationship between brace prescription and brace wear [17]. Our data showed that on average, the longer the brace was prescribed, the greater the deficiency between prescribed hours and hours worn. This may be relevant to patients who are at higher risk of progression such as more skeletally immature patients with larger curves. As such, a longer brace prescription may be more critical in this subgroup. The raw data of our study demonstrated diminishing returns for this effect with brace prescriptions longer than 16 h.

In the present study, there was a significant difference in the number of hours prescribed to be in the brace and the actual number of hours the brace was worn measured by the heat sensor. The prescription time was consistently greater than the actual hours worn. The variability in these findings elucidate the need for a better understanding of optimal time prescribed and brace adherence for each individual patient to maximize compliance and minimize surgical conversion.

Comparable to the findings of Weinstein’s BRAIST study and Katz’ observation trial, we found higher curve stability in those who wore the brace longer [21, 25]. The BRAIST study found that >12.9 h of bracing had 90–93% success rates compared to < 6 h having similar success rates to observation alone [25]. In our analysis, 86.7% of patients who wore the brace for 12 or more hours per day had a successful outcome defined by a curve progression <6° compared to 76.6% of patients who wore the brace <12 h per day. Although not the specific question our study was designed to answer, our data suggest a similar dose-dependent relationship with brace prescription, brace compliance, and curve progression, though our result was not statistically significant.

There were several limitations to this study. Data were prospectively collected; however, many patients did not get heat sensor monitor data. Patients were excluded if they were missing this adherence data. The heat sensor technology is subject to errors in longevity and compatibility, decreasing the number of patients in our cohort with bracing data. Furthermore, the short follow-up period is another limitation within this study, minimizing the final outcome of patient’s clinical course once reaching skeletal maturity evidenced by a Sanders score of 7b. However, the longitudinal, prospective nature of the study design will follow these results to fruition and do not impact the questions we sought to answer in this study. Excluding patients who were undergoing nighttime bracing or <12 h/day may also be viewed as a limitation. However, this was done to remove potentially confounding effects of a different bracing philosophy (nighttime hyper-correction braces). Further longitudinal data may shed light on brace adherence throughout the course of treatment and answer questions such as if adherence levels off with time, if it improves or get worse, and what risk factors can help predict this relationship.

Strengths of our study include the prospective, multicenter design. As our study is a multicenter investigation, the results reflect the outcomes of a variety of surgeons’ treatment plans across institutions allowing for generalizability of our data. The standardized, objective monitoring of brace wear is another strength of our study.

In conclusion, patients consistently wear the brace less than prescribed. The average rate of nonadherence increases as the number of hours prescribed increases. Patients wear the brace on average 4 h less than prescribed. As prescribed hours were the only modifiable risk factor identified, our focus should likely be on optimizing that variable. As such, providers should consider prescribing at least 16 h per day of brace wear for skeletally immature patients at risk of progression with the intention of optimizing the chance to wear the brace greater than 12 h daily.

## Data Availability

The data are not publicly available due to privacy restrictions. The data are available from the corresponding author on reasonable request.
